# Supplementary material for: The mitochondrial methylation potential gates mitoribosome assembly
Source: Nat Commun. 2025 Jun 25;16:5388. doi: 10.1038/s41467-025-60977-x (PMC12198368; doi:10.1038/s41467-025-60977-x)
Supplement: Supplementary file 1 — Supplementary Information [file 41467_2025_60977_MOESM1_ESM.pdf]

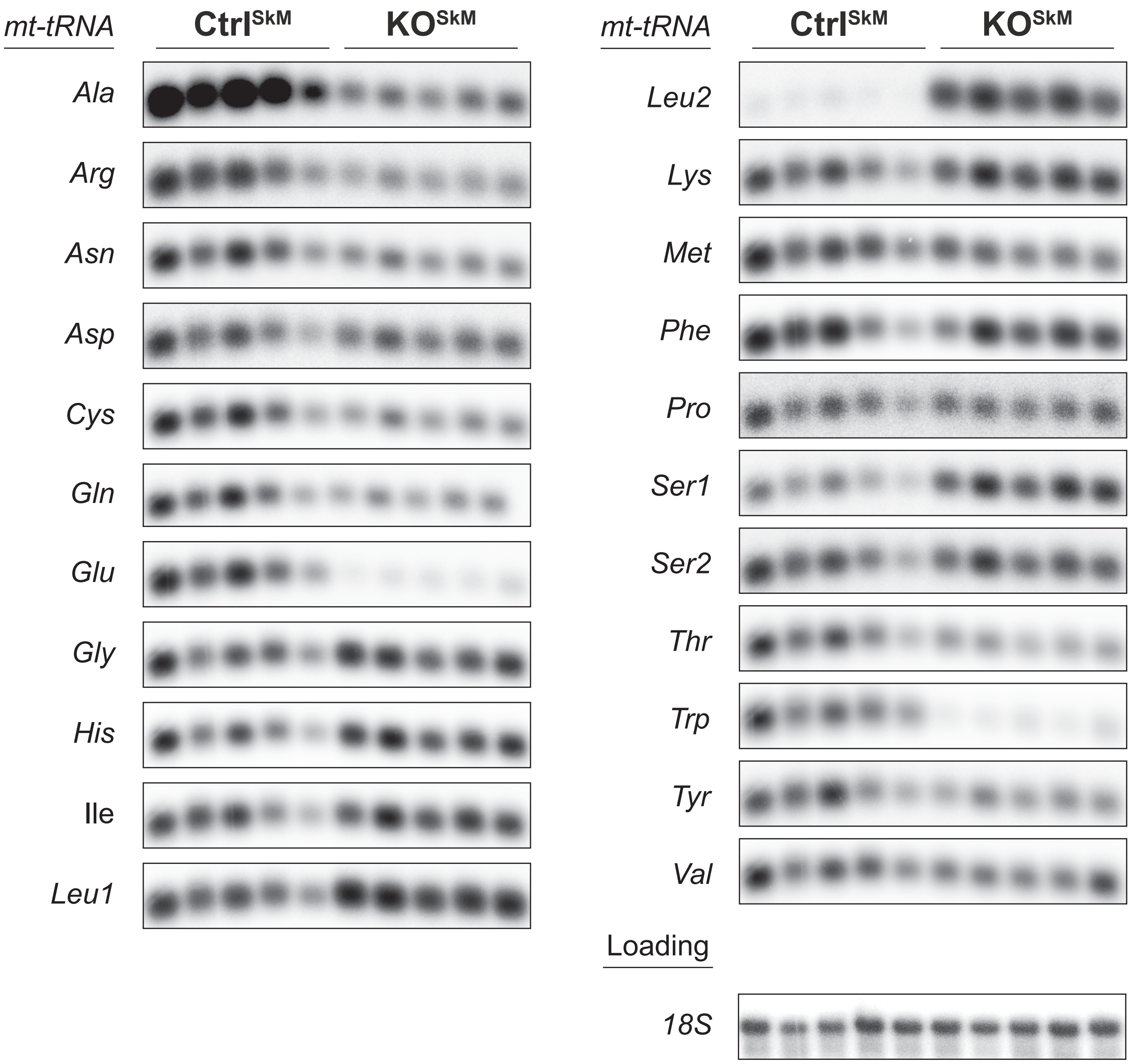

Supplementary Fig. 1. Steady-state levels of mt-tRNAs in skeletal muscle
mt-tRNA steady-state levels determined by Northern blot analysis in quadriceps from control (Ctrl^{SkM}) and *Samc* KO (KO^{SkM}) mice at 12 weeks of age. Oligo probes were used against all mitochondrial encoded tRNAs and 18S rRNA was probed as loading control. (N=5 independent samples per genotype) Source data are provided as a Source Data file.

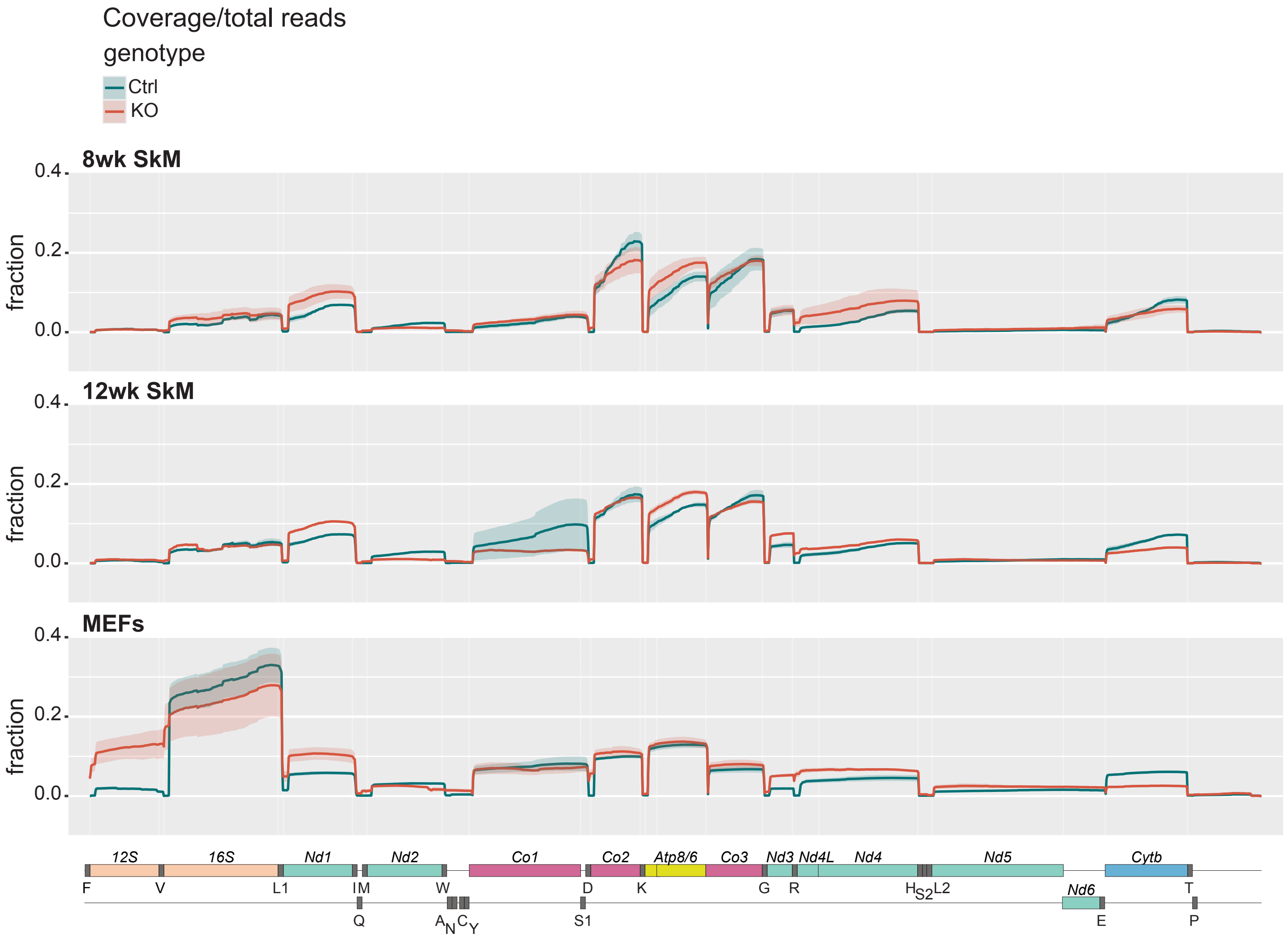

Supplementary Fig. 2. Coverage of ONT sequencing

Coverage of ONT RNA sequencing across the mitochondrial genome in *Samc* KO and control skeletal muscle samples at 8 and 12 weeks of age and MEFs. Coverage caluclated as a fraction of total reads, with mean coverage plotted for KO (dark red) and Control (dark green). The data spread of three replicates plotted for KO (light red) and control (light green) are shown. (N=3 independent samples per sample and genotype)

a

Transcript boundaries used.

Transcript	start	End
mtRnr1 (12S)	70	1024
mt-Rnr2 (16S)	1094	2675
mtNd1	2751	3707
mtNd2	3914	4951
mtCo1	5327	6941
mtCo2	7012	7699
mtAtp8/6	7765	8606
mtCo3	8607	9390
mtNd3	9459	9807
mtNd4/4L	9876	11545
mtNd5	11742	14133
mtNd6	14070	12405
mtCytb	14140	15288

b

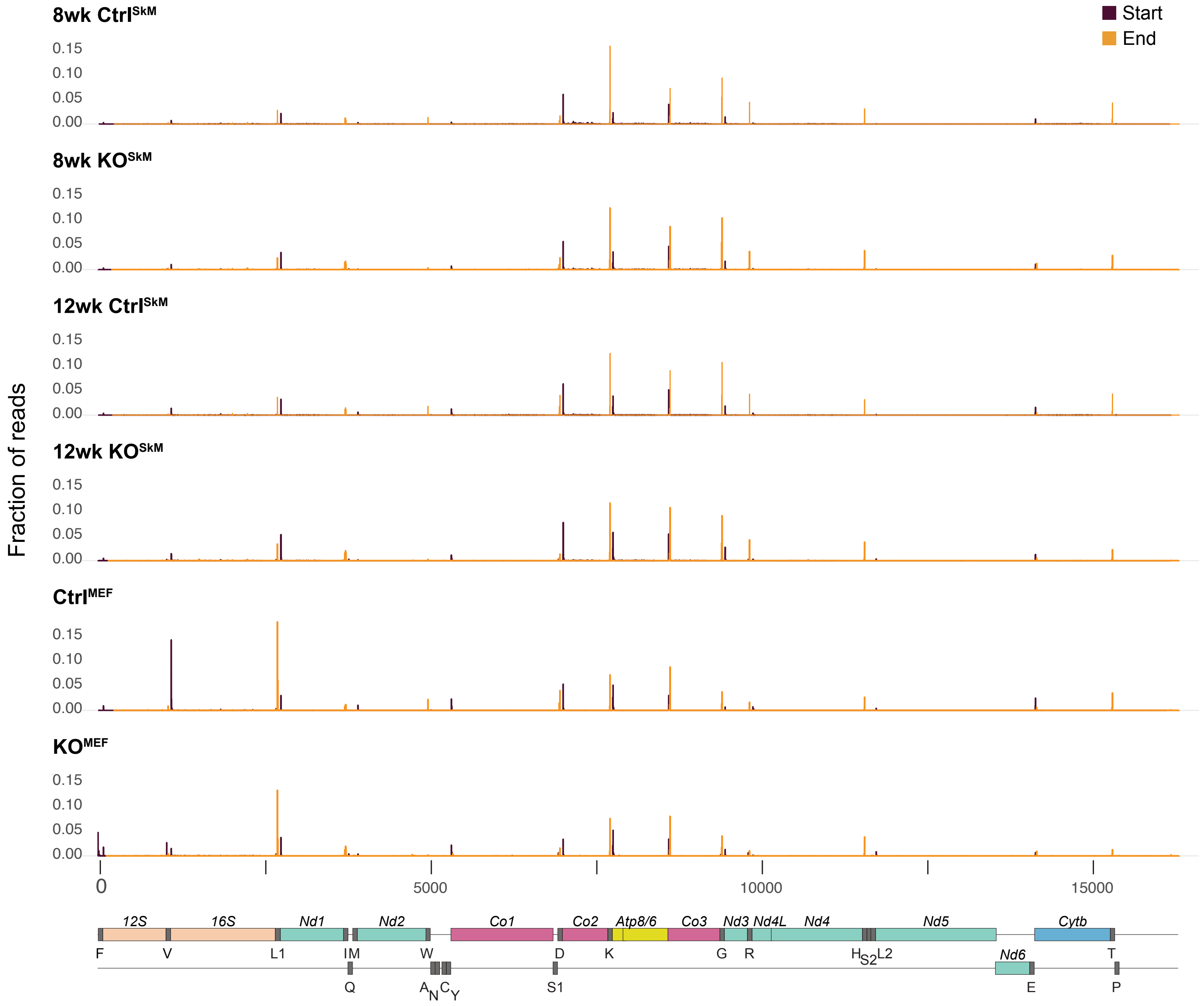

Supplementary Fig. 3. Start and end sites of total ONT sequencing reads.

(**a**) Transcript boundaries were defined accodring to gene boundaries described in the mouse mtDNA reference sequecne (NC005089), modified with untranslated regions descibed in Temperely et al. (2010) (PMID:20211597). (**b**) All start (plumb) and end (orange) sites from total ONT sequencing data, calculated as a fraction of total reads and aligned to the mitochondrial genome in KO and control samples in quadriceps of 8- and 12-week-old mice and MEFs.

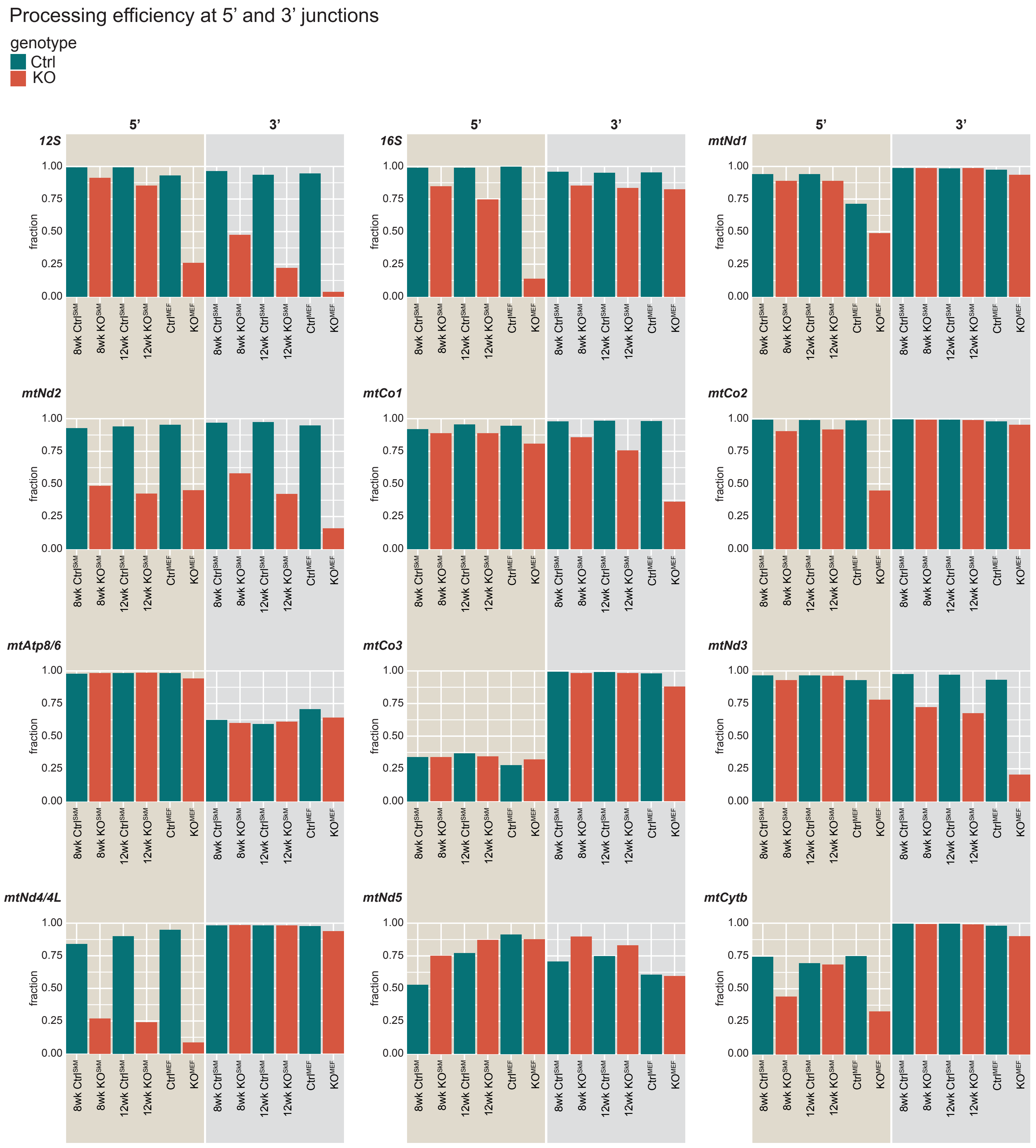

Supplementary Fig. 4. Junction-specific processing efficiencies
Processing efiiciency at all heavy-strand encoded rRNA and mRNA junctions from ONT sequencing of KO (red) and control (green) samples in 8 and 12 week mice and MEFs. Efficiency plotted as fraction of fully processed reads at 5′ (beige panels) or 3′ (grey panels) per transcript.

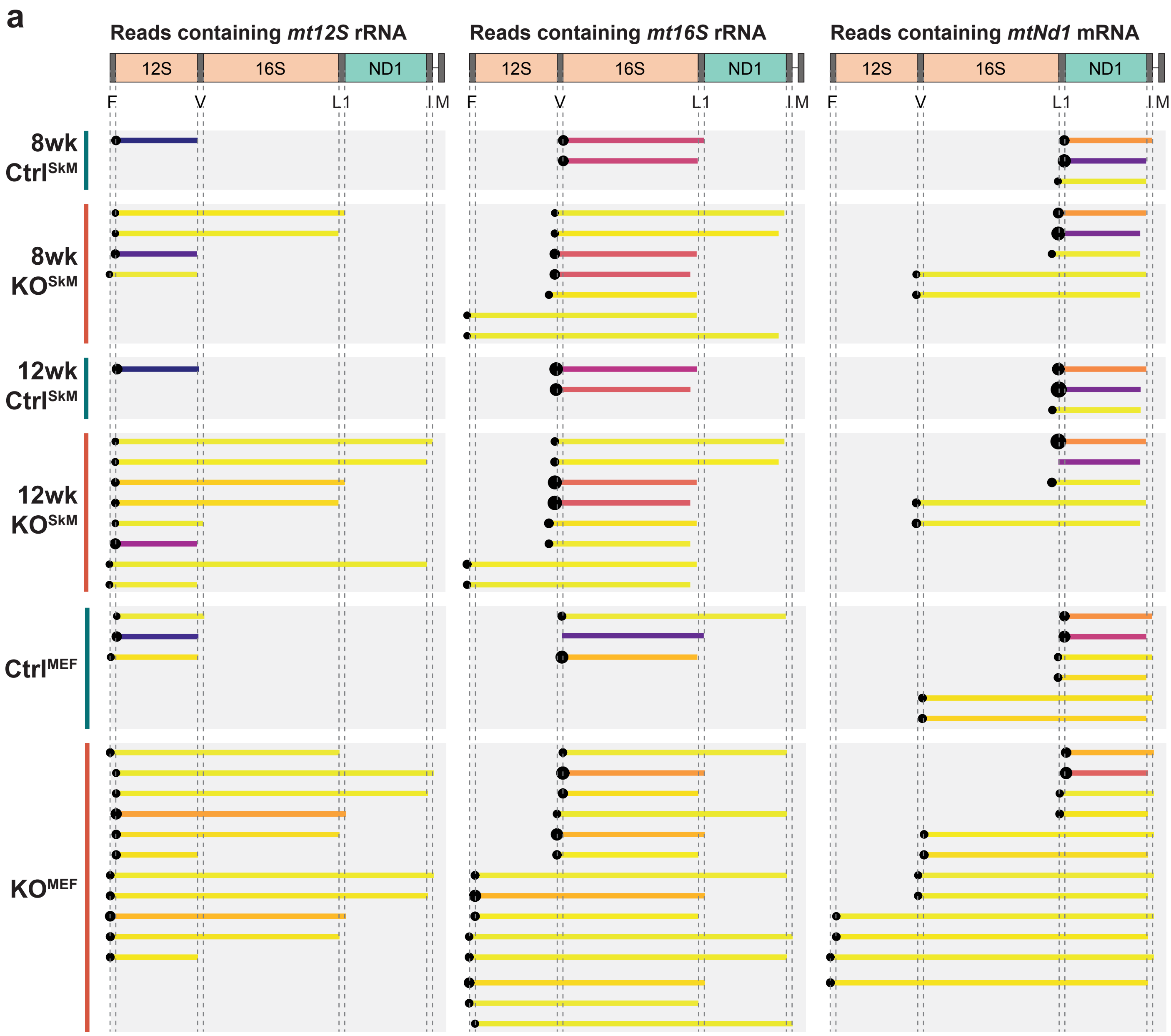

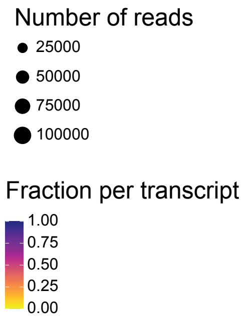

b

% of processed transcripts in the rRNA cluster region

	8wk		12wk			
	$Ctrl^{SkM}$	KO^{SkM}	$Ctrl^{SkM}$	KO^{SkM}	$Ctrl^{MEF}$	KO^{MEF}
12S	98,3	85.6	98,7	63,5	91,4	5,8
16S	49,9	43,5	41,9	42,5	14,9	5,9
Nd1	79,1	74,7	74,4	68,5	52,2	40,0

Supplementary Fig. 5. Processing intermediates at the rRNA cluster

a) Start and end sites of all reads containing *mt12S* rRNA (left panel), *mt16S* rRNA (middle panel) and *mtNd1* mRNA (right panel) in KO and control samples in 8 and 12 week mice and MEFs. Fraction cut-off for reads plotted set at ≥0.01. Canonical processing boundaries depicted with dashed lines. **b**) Percentage of completely processed (at both 5' and 3' ends) 12S (RNR1), 16S (RNR2) and ND1 trancripts within each sample group.

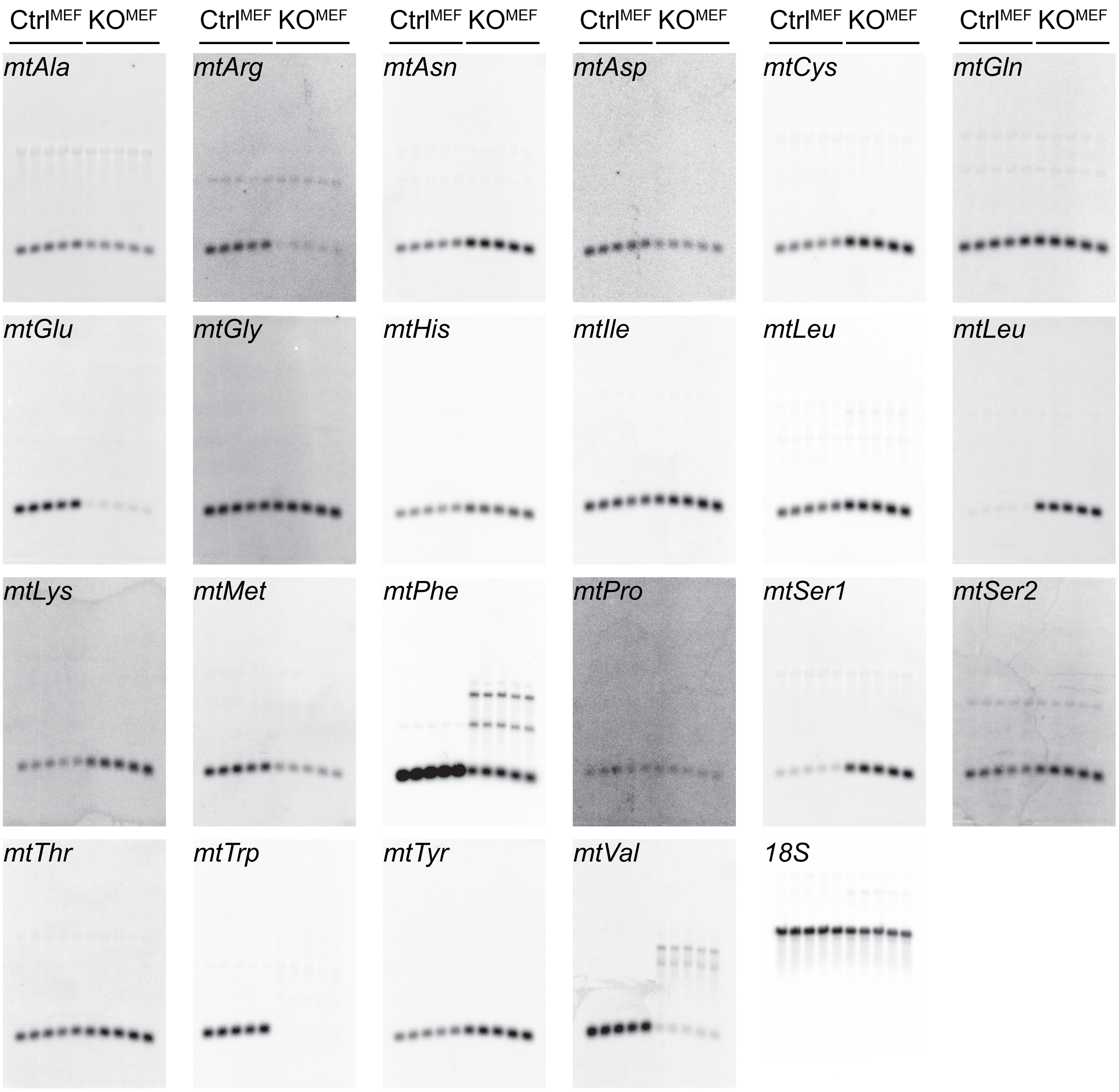

Supplementary Fig. 6. Full-length Northern blots of mitochondrial tRNA steady-state levels

Northern blot analysis of control (Ctrl) and *Samc* KO MEFs, using probes against mt-tRNAs as indicated. Blots displayed in main Figure 1d are represented here in an un-cropped format to demonstrate the presence of larger species when probing for a subset of mt-tRNAs in the KO[MEF] samples. 18S is probed as loading control. (N=5 independent samples per genotype) Source data are provided as a Source Data file.

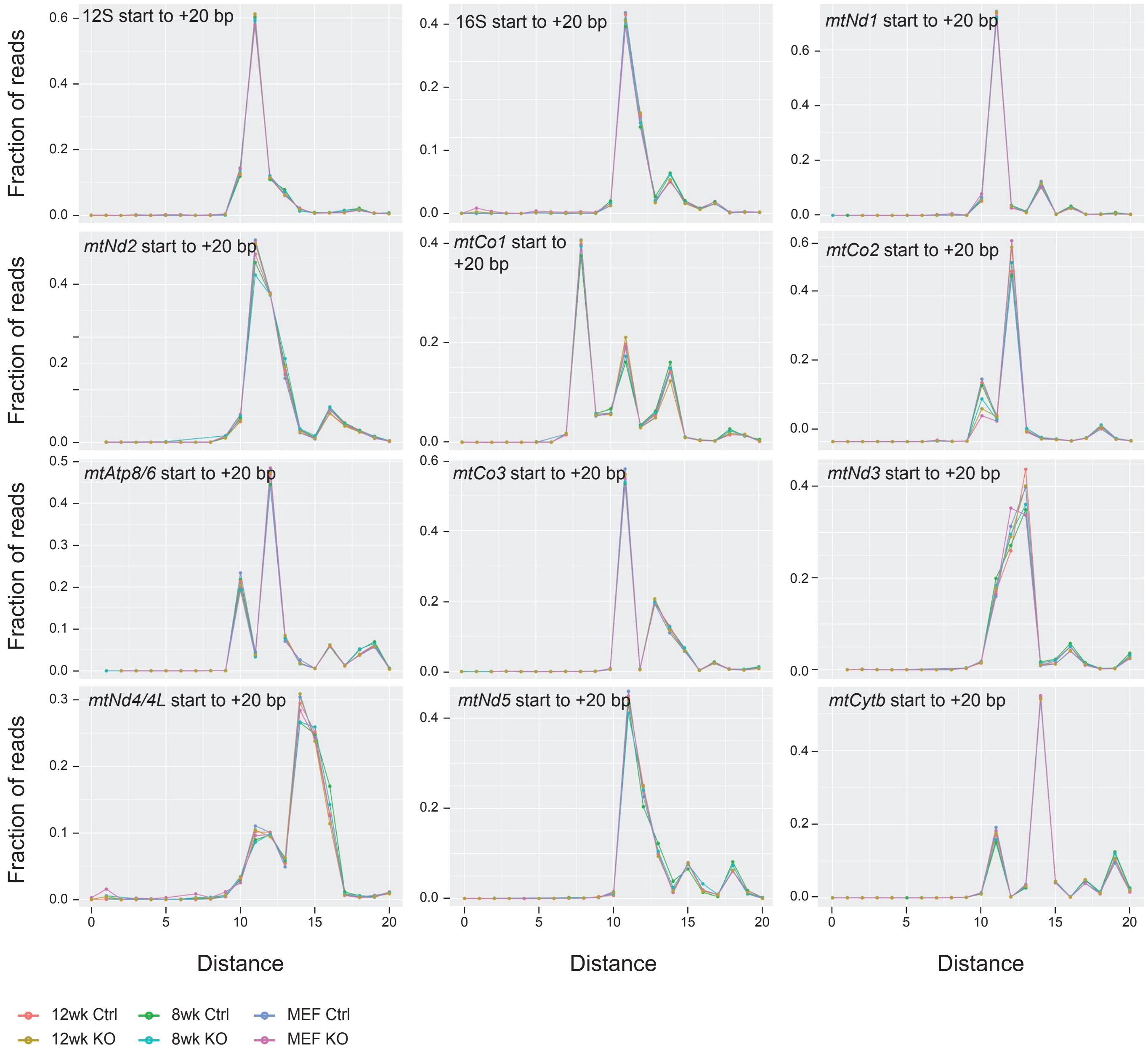

Supplementary Fig. 7. Start site frequency determined by ONT

Start site frequency of fully processed transcripts in ONT sequencing data in samples from 8 week mice (control orange, *Samc* KO yellow), 12 week mice (control green, *Samc* KO turquoise) and MEFs (control blue, *Samc* KO pink). Distance from the annotated start site shown up to +20 bp.

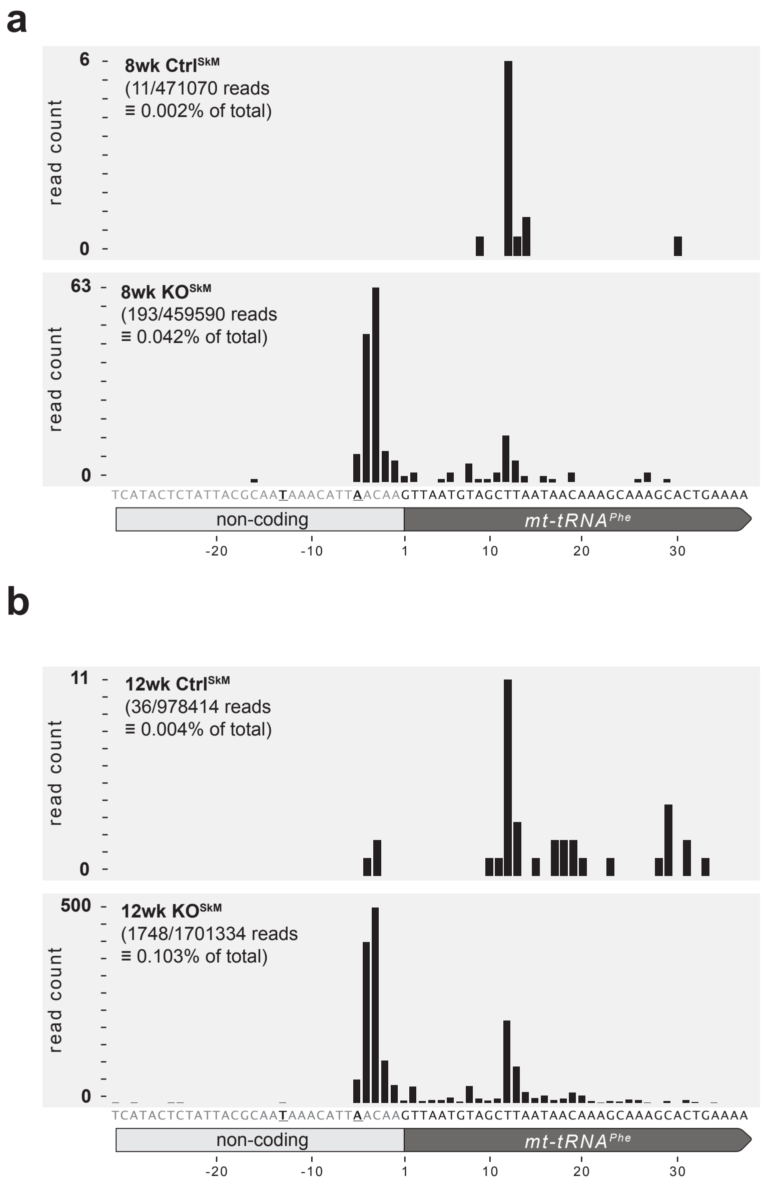

Supplementary Fig. 8. Start site frequency at mt-tRNA-Phe

Number of transcription start sites for reads containing mt-tRNA-Phe, as detected by ONT analysis in quadriceps preparations from (**a**) 8-week-old and (**b**) 12-week-old animals. The 5′ leader sequence is shown in grey, with transcription initiation sites highlighted in bold and underlined. Due to technical limitations of ONT sequencing, accurate 5′ end sequencing is not possible, resulting in the loss of approximately 12 nucleotides.

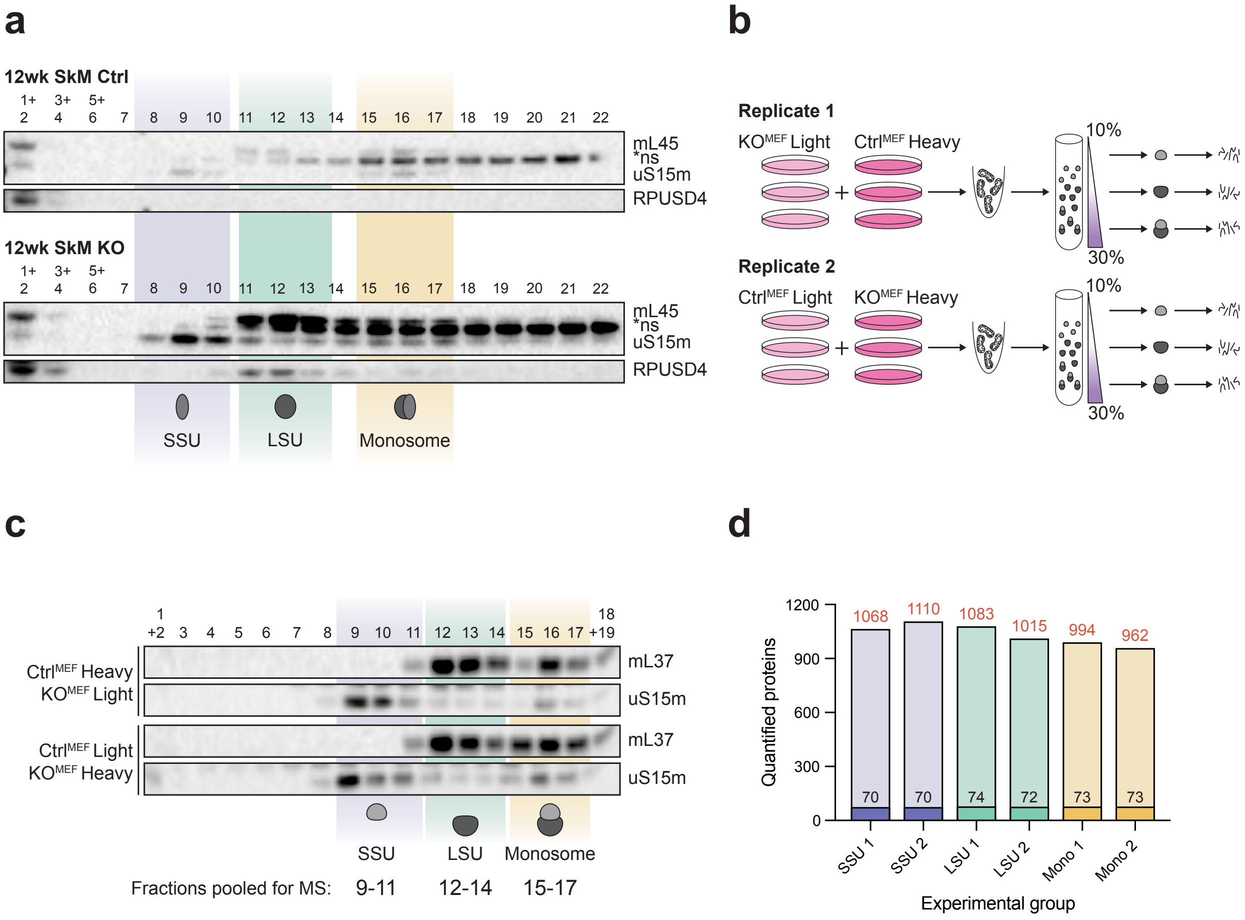

Supplementary Fig. 9. Ribosome assembly analysis.

(**a**) Western blot analysis of sucrose gradient fractions from 12-week-old quadriceps mitochondrial preparations from KO and control mice, probed with the indicated antibodies. ns = non-specific band. **(b)** Schematic representation of the experimental design for SILAC labeling. Two replicates were run in parallel with inverse stable isotope labeling, using heavy lysine [$^{13}C_6$,$^{15}N_2$] and heavy arginine [$^{13}C_6$,$^{15}N_4$] in either KO or control MEFs. After labelling, KO and control mitochondria were enriched and merged, followed by sucrose gradient sedimentation. Peptides from pooled fractions corresponding to the mitoribosomal small subunit, large subunit, and monosome were analysed by mass spectrometry (MS). (**c**) Western blot analysis of merged KO and control samples following SILAC labeling and sucrose gradient sedimentation. Samples were probed with antibodies for the large mitoribosomal subunit (mL37) and the small mitoribosomal subunit (uS15m) to confirm sedimentation patterns for fraction pooling. (**d**) Number of peptides identified per sample. The total number of peptides is shown in red, while mitoribosome-specific peptides (dark) are shown in black. Source data are provided as a Source Data file.

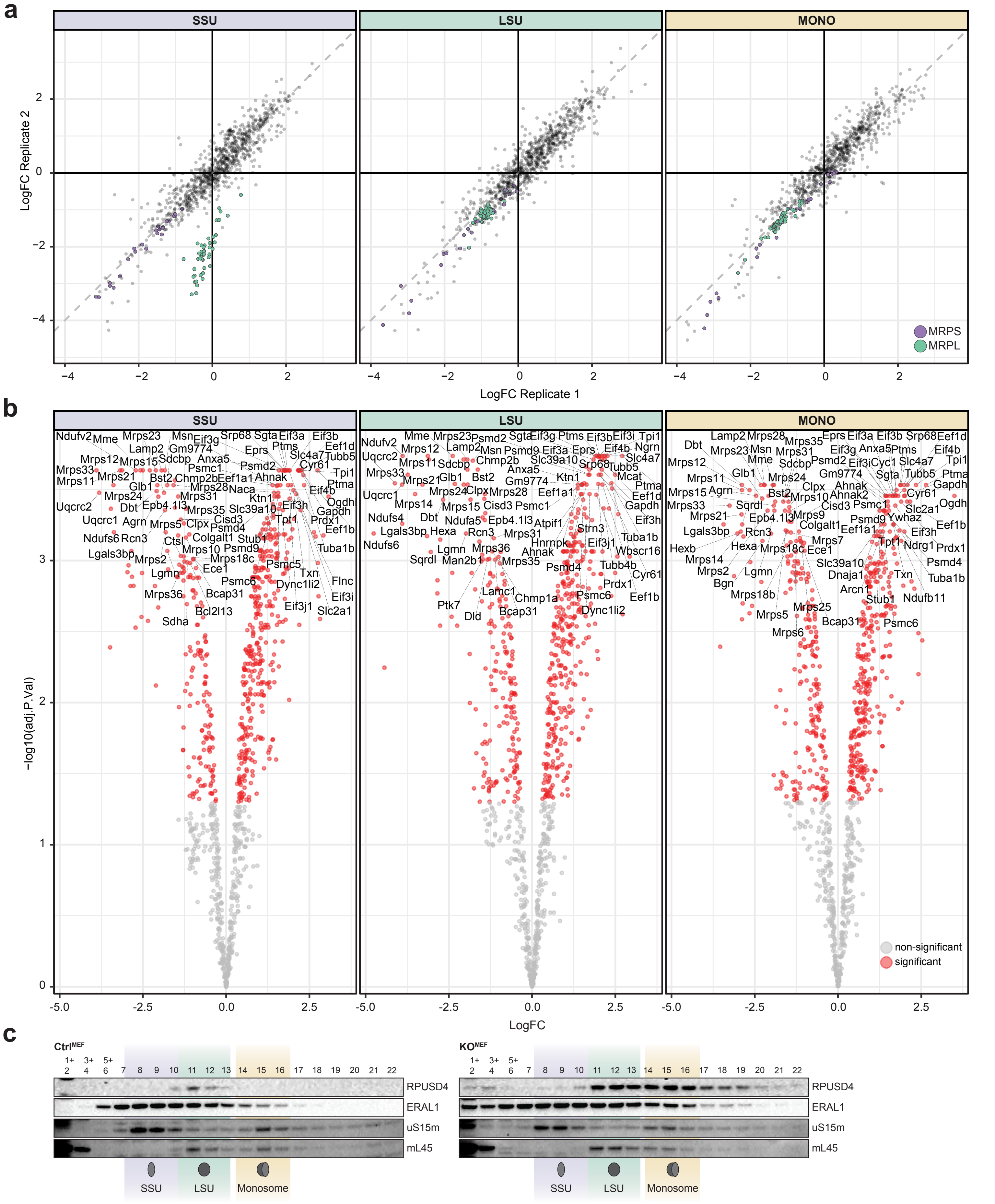

Supplementary Fig. 10. Ribosome profiling using SILAC

(**a**) Quantitative reproducibility of two independent SILAC proteome replicates, plotting KO vs. Control logFC in mtSSU, mtLSU, and monosome fraction pools. Proteins of the mtSSU (purple) and mtLSU (green) are highlighted. (**b**) Volcano plot presenting the total SILAC proteome KO vs. Control logFC in mtSSU, mtLSU, and monosome fraction pools. Proteins with a significant adjusted P-value are shown in red. The 40 proteins with the most positive and negative fold-changes are labeled in each fraction pool. (**c**) Western blot analysis of sucrose gradient fractions from KO and control MEFs. Early fractions were merged (1+2, 3+4, 5+6), and samples were loaded onto wide 20-well SDS-PAGE gels to visualise late fractions (20–22). Gels were overloaded to ensure visualisation of low expressed proteins. Blots were decorated with antibodies against proteins enriched in monosome fractions in the SILAC proteome of KO^{MEFs}. Source data are provided as a Source Data file.

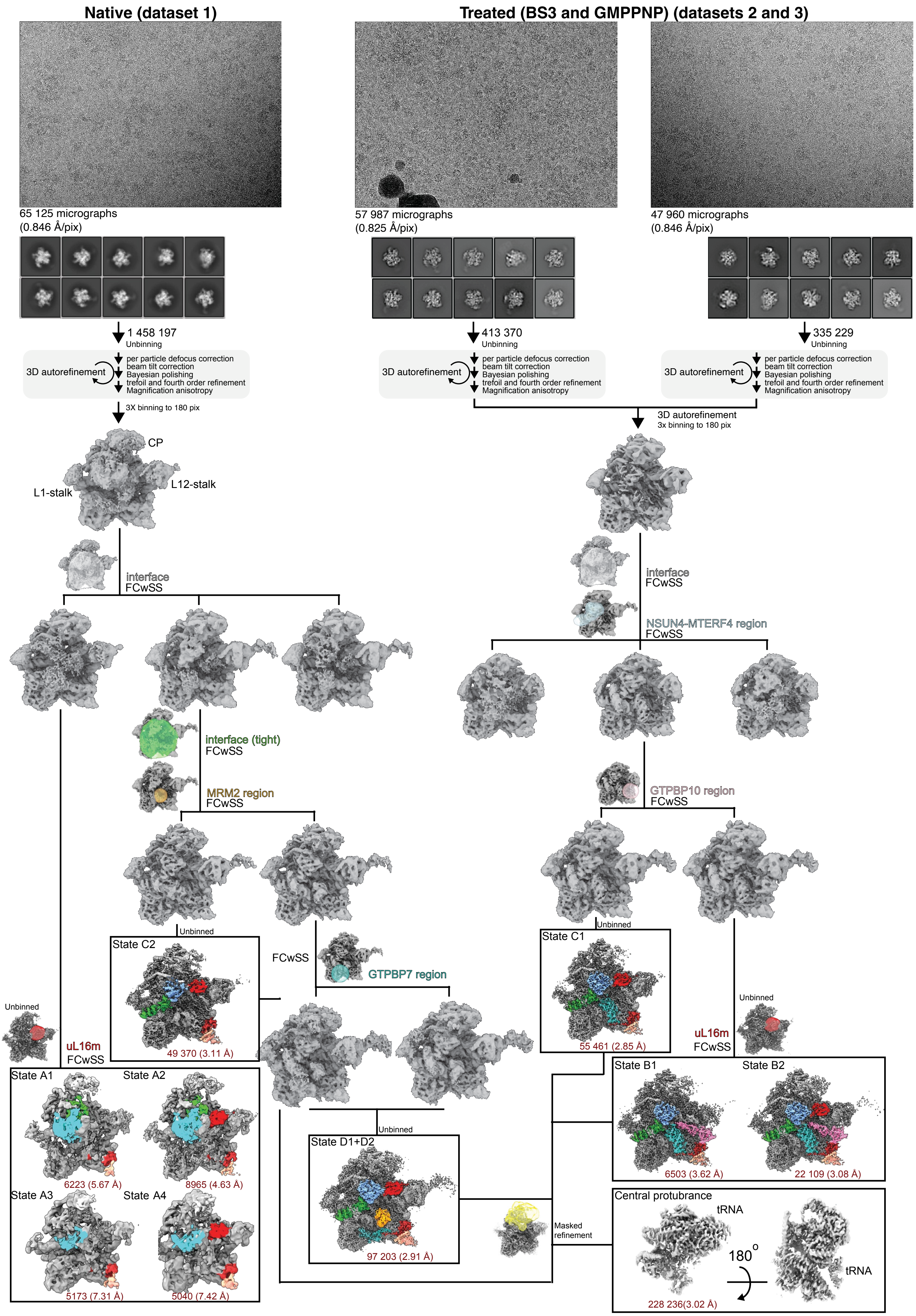

Supplementary Fig. 11. Cryo EM data processing scheme.

Top panel: Representative motion-corrected micrographs followed by representative 2D class averages.

Lower panels: Schematic representation of the focused 3D classification with signal subtraction (FCwSS) strategy used to resolve structural heterogeneity. Final unbinned maps are boxed, with their respective particle numbers and resolutions indicated in red. Resolutions were estimated using Fourier Shell Correlation (FSC) at a 0.143 cut-off between half-maps.

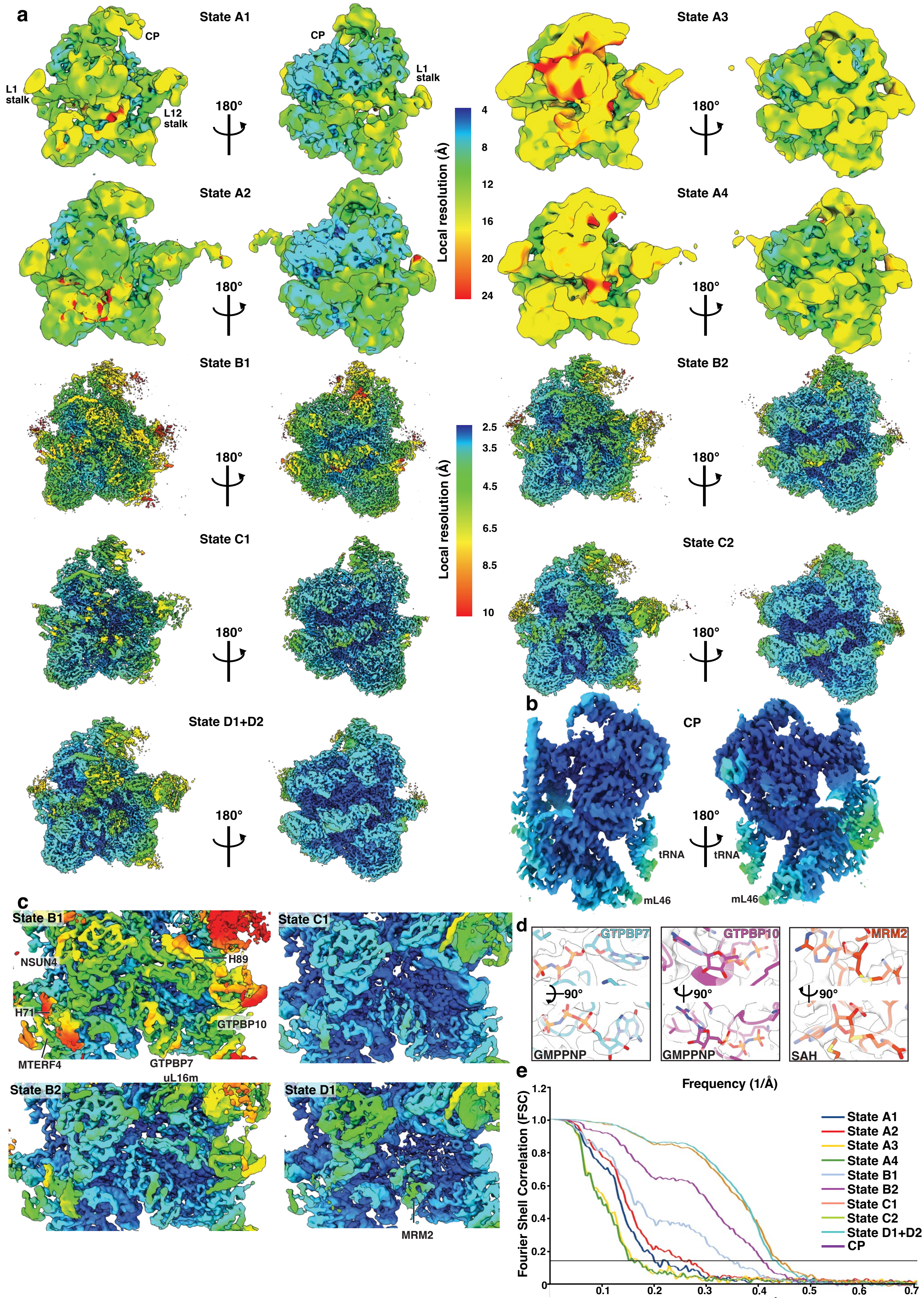

Supplementary Fig. 12. Local resolution maps

(**a**) Overall maps of all mtLSU assembly states, coloured by local resolution. The corresponding colour key indicates the resolution range, with separate scales applied for states A1–A4 and B1–D2. (**b**) Masked refined CP map from particled pooled from states B-D coloured by local resolution scaled according states B-D. (**c**) Masked refined PTC map of individual states B-D coloured by local resolution scaled according states B-D. (d) Panels show model-map fit for ligands GMPPNP (bound to GTPBP7 and GTPBP10) and SAH (bound to MRM2) from states C1, B2 and D, respectively. (**e**) Fourier Shell Correlation (FSC) curves between half-maps for all states, with the 0.143 threshold marked by a black line.

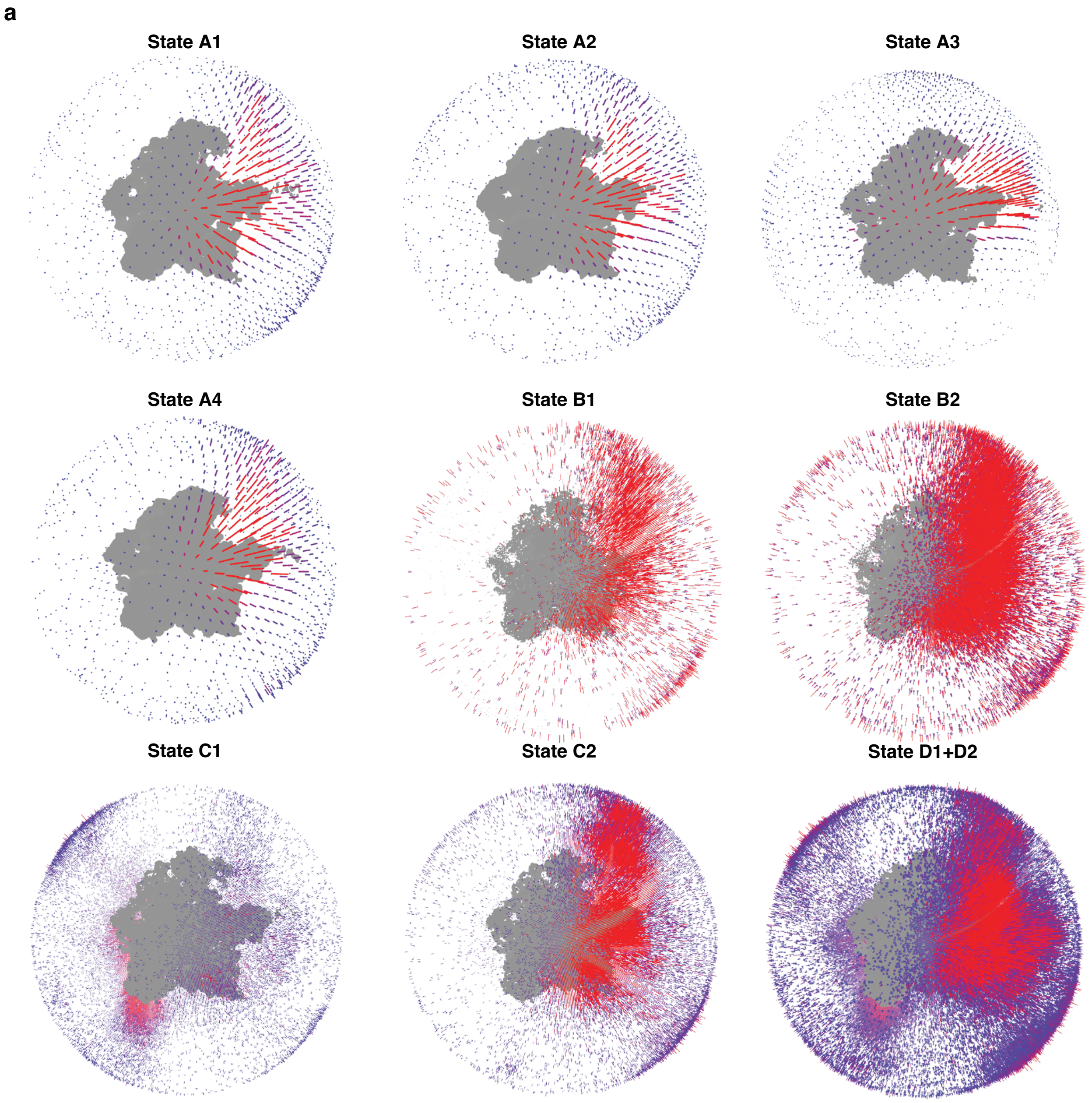

Supplementary Fig. 13. Figure shows Euler angle distribution of aligned particle images for all states superposed against corresponding maps (grey).

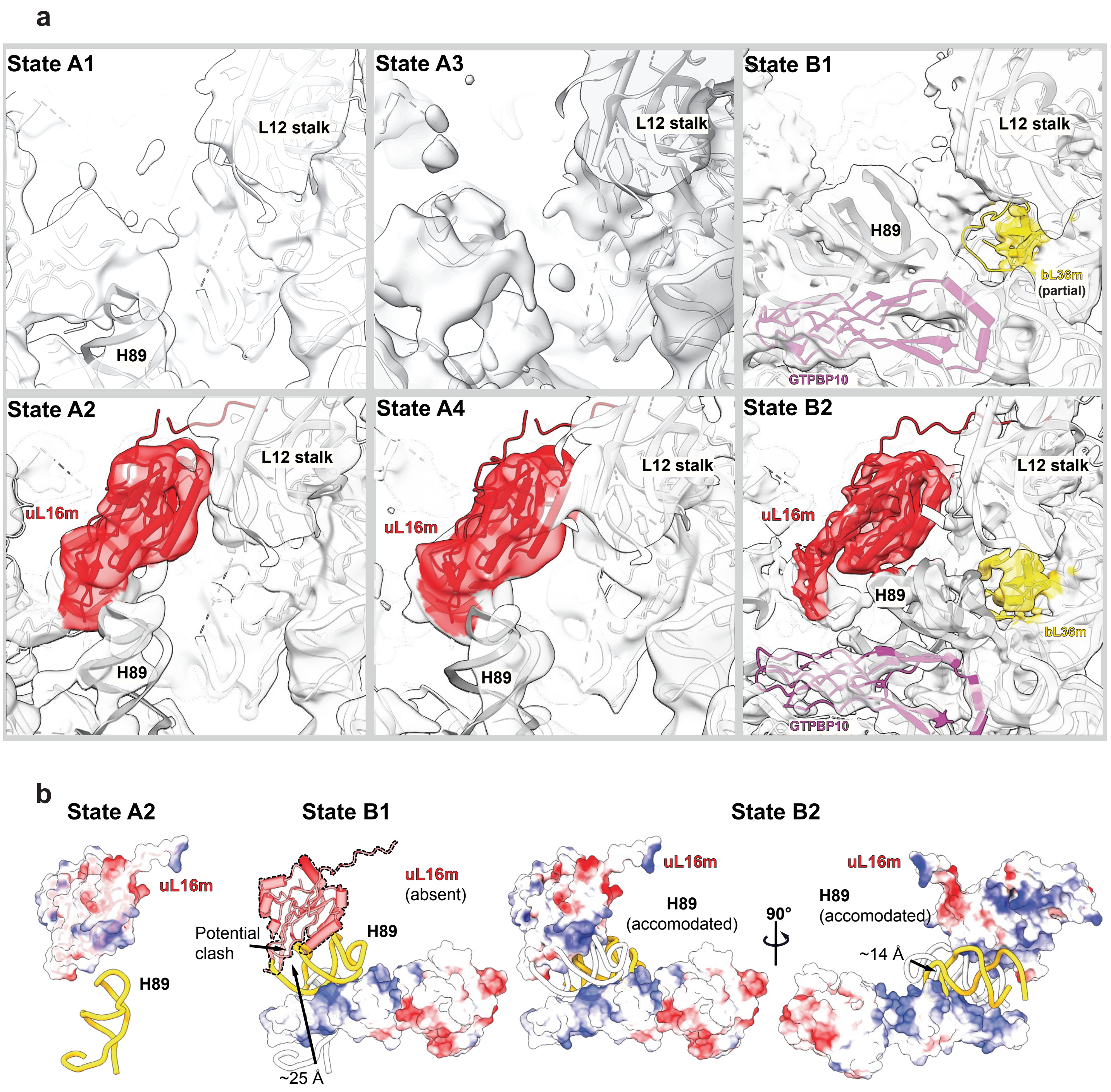

Supplementary Fig. 14. Accommodation of uL16m, bL36m, and maturation of H89

(**a**) States A1–A4 (left and centre) show unstable binding of uL16m, while bL36m is entirely absent. In states A2 and A4, H89 is modelled into a weak density near uL16m but becomes increasingly disordered in its absence (A1 and A3). In contrast, states B1 and B2 (right) feature the binding of GTPBP10. In state B1, uL16m is absent, while bL36m appears for the first time at partial occupancy, as indicated by weak density. In state B2, both uL16m and bL36m bind stably, and H89 adopts its final conformation. (**b**) Panels illustrate the conformational rearrangement of H89 from A2 (left) to its mature state in B2 (right), guided by uL16m and GTPBP10 (colored by electrostatic potential). In each panel, the current conformation of H89 (gold) is compared to its previous state (white). The absence of uL16m in state B1 is highlighted by a dotted outline.
