# Supplementary material for: The mitochondrial methylation potential gates mitoribosome assembly
Source: Nat Commun. 2025 Jun 25;16:5388. doi: 10.1038/s41467-025-60977-x (PMC12198368; doi:10.1038/s41467-025-60977-x)
Supplement: Supplementary file 2 — Description of Additional Supplementary Files [file 41467_2025_60977_MOESM2_ESM.pdf]

Description of additional Supplementary Data Files

File name: Supplementary Data 1

Description: SkM and MEF proteomics KO Vs Ctrl. Processed proteomics data from 8 and 12-week-old skeletal muscle and MEF samples.

File name: Supplementary Data 2

Description: Quality control and read counts from direct RNA long-read Oxford nanopore sequencing of RNA from 8 and 12-week-old skeletal muscle and MEF samples.

Fine name: Supplementary Data 3
Description: Oxford nanopore sequencing data of RNA from control MEFs.

File name: Supplementary Data 4
Description: Oxford nanopore sequencing data of RNA from KO MEFs.

File name: Supplementary Data 5
Description: Oxford nanopore sequencing data of skel. muscle RNA from 8-week-old control mice.

File name: Supplementary Data 6
Description: Oxford nanopore sequencing data of skel. muscle RNA from 8-week-old SAMC KO mice.

File name: Supplementary Data 7
Description: Oxford nanopore sequencing data of skel. muscle RNA from 12-week-old control mice.

File name: Supplementary Data 8
Description: Oxford nanopore sequencing data of skel. muscle RNA from 12-week-old SAMC KO mice.

File name: Supplementary Data 9
Description: Oxford nanopore sequencing depth summary from 8- and 12-week-old skeletal muscle and MEF samples.

File name: Supplementary Data 10
Description: MEF proteome KO Vs Ctrl. Processed proteomics data from SILAC labelled ribosome gradient fractions of cotronl and KO MEF samples.

File name: Supplementary Data 11
Description: List of antibodies and primers used in this study

File name: Supplementary Data 12
Description: Information regarding Cryo-EM collection settings, refinement and validation statistics.
