# Supplementary material for: The mitochondrial methylation potential gates mitoribosome assembly
Source: Nat Commun. 2025 Jun 25;16:5388. doi: 10.1038/s41467-025-60977-x (PMC12198368; doi:10.1038/s41467-025-60977-x)
Supplement: Supplementary file 12 — Reporting Summary [file 41467_2025_60977_MOESM12_ESM.pdf]

nature portfolio

Corresponding author(s): Anna Wredenberg

Last updated by author(s): Apr 5, 2025

Reporting Summary

Nature Portfolio wishes to improve the reproducibility of the work that we publish. This form provides structure for consistency and transparency in reporting. For further information on Nature Portfolio policies, see our Editorial Policies and the Editorial Policy Checklist.

Statistics

For all statistical analyses, confirm that the following items are present in the figure legend, table legend, main text, or Methods section.

n/a	Confirmed	
☐	☒	The exact sample size (*n*) for each experimental group/condition, given as a discrete number and unit of measurement
☐	☒	A statement on whether measurements were taken from distinct samples or whether the same sample was measured repeatedly
☐	☒	The statistical test(s) used AND whether they are one- or two-sided<br>*Only common tests should be described solely by name; describe more complex techniques in the Methods section.*
☒	☐	A description of all covariates tested
☒	☐	A description of any assumptions or corrections, such as tests of normality and adjustment for multiple comparisons
☒	☐	A full description of the statistical parameters including central tendency (e.g. means) or other basic estimates (e.g. regression coefficient) AND variation (e.g. standard deviation) or associated estimates of uncertainty (e.g. confidence intervals)
☒	☐	For null hypothesis testing, the test statistic (e.g. *F*, *t*, *r*) with confidence intervals, effect sizes, degrees of freedom and *P* value noted<br>*Give P values as exact values whenever suitable.*
☒	☐	For Bayesian analysis, information on the choice of priors and Markov chain Monte Carlo settings
☒	☐	For hierarchical and complex designs, identification of the appropriate level for tests and full reporting of outcomes
☒	☐	Estimates of effect sizes (e.g. Cohen's *d*, Pearson's *r*), indicating how they were calculated

Our web collection on statistics for biologists contains articles on many of the points above.

Software and code

Policy information about availability of computer code

Data collection: No code was utilised during data collection.

Data analysis:

Nanopore data analysis
No novel code or software developed.
Fast5 files were merged using the multi_to_single_fast5 function from the ont_fast5_api toolkit (https://github.com/nanoporetech/ont_fast5_api/tree/master). The merged files were then re-basecalled using Guppy v4.4.1 with the following parameters: --flowcell FLO-MIN106, --kit SQK-RNA002, --recursive, --fast5_out, and --qscore_filtering 7. The resulting FASTQ files were mapped to the mitochondrial genome in the GRCm38 reference using Minimap2 v2.17 with parameters: -ax splice -uf -k1459. The SAM files generated from this mapping were converted to BAM format, then sorted and indexed using Samtools v1.1060. Next, the BAM files were converted to BED format using the bamtobed function from Bedtools v2.29.261. The resulting BED files with same age and genotype were merged, processed, and visualised in R v4.3.1 using the ggplot2 package v3.4.3 for visualisation.

LC/MS data analysis
No novel code or software developed.
The raw data were analysed with MaxQuant version 1.6.1.063. Peptide fragmentation spectra were searched against the canonical and sequences of the Mus musculus reference proteome (proteome ID UP000000589, downloaded December 2018 from UniProt).

For manuscripts utilizing custom algorithms or software that are central to the research but not yet described in published literature, software must be made available to editors and reviewers. We strongly encourage code deposition in a community repository (e.g. GitHub). See the Nature Portfolio guidelines for submitting code & software for further information.

Data

Policy information about availability of data

All manuscripts must include a data availability statement. This statement should provide the following information, where applicable:

- Accession codes, unique identifiers, or web links for publicly available datasets
- A description of any restrictions on data availability
- For clinical datasets or third party data, please ensure that the statement adheres to our policy

The mass spectrometry proteomics data have been deposited to the ProteomeXchange ConsorXum via the PRIDE partner repository (https://www.ebi.ac.uk/pride/) with the dataset idenXfier PXD055907; username: reviewer_pxd055907@ebi.ac.uk, password: 7T40I9jP6fq9 and idenXfiler PXD062614; username: reviewer_pxd062614@ebi.ac.uk; password: nUX8kfpdFwsB.

The ONT RNA sequencing data have been deposited at NCBI SRA and can be viewed at:
https://dataview.ncbi.nlm.nih.gov/object/PRJNA1192297?reviewer=hn69if5m2j7se8sf43jgdl2cs6

Research involving human participants, their data, or biological material

Policy information about studies with human participants or human data. See also policy information about sex, gender (identity/presentation), and sexual orientation and race, ethnicity and racism.

Reporting on sex and gender	N/A
Reporting on race, ethnicity, or other socially relevant groupings	N/A
Population characteristics	N/A
Recruitment	N/A
Ethics oversight	N/A

Note that full information on the approval of the study protocol must also be provided in the manuscript.

Field-specific reporting

Please select the one below that is the best fit for your research. If you are not sure, read the appropriate sections before making your selection.

☒ Life sciences ☐ Behavioural & social sciences ☐ Ecological, evolutionary & environmental sciences

For a reference copy of the document with all sections, see nature.com/documents/nr-reporting-summary-flat.pdf

Life sciences study design

All studies must disclose on these points even when the disclosure is negative.

Sample size	Sample size for most experiments was n=3 per genotype/experimental group, chosen for statistical power in t tests and based on consistent results within early characterization studies of the mouse model. Each experimental sample size is listed in the figure legend of every figure.
Data exclusions	No data exclusions
Replication	All experiments were replicated between 2 and 5 times with reproducible results, with the exception of one experiment outlined in Figure 4d.
Randomization	No randomisation was relevant to the study. Cell lines and tissue samples were taken from animals selected due to a specific genotype.
Blinding	Blinding not necessary because experimental group allocation was based on genotype alone.

Reporting for specific materials, systems and methods

We require information from authors about some types of materials, experimental systems and methods used in many studies. Here, indicate whether each material, system or method listed is relevant to your study. If you are not sure if a list item applies to your research, read the appropriate section before selecting a response.

Materials & experimental systems

n/a	Involved in the study	
	☒	Antibodies
	☒	Eukaryotic cell lines
☒		Palaeontology and archaeology
	☒	Animals and other organisms
☒		Clinical data
☒		Dual use research of concern
☒		Plants

Methods

n/a	Involved in the study	
☒		ChIP-seq
☒		Flow cytometry
☒		MRI-based neuroimaging

Antibodies

Antibodies used

Primary Antibodies
Target Manufacturer Catalog No.
MRPS22 (mS22) Thermo Fisher 10984-1-AP
MRPL28 (bL28m) Sigma Prestige Antibodies HPA030594
MRPS15 (uS15m) Proteintech 17006-1-AP
MRPL45 (mL45 Proteintech 15682-1-AP
MRPS16 (bS16m) Sigma HPA050081
MRPS35 (mS35) Proteintech 16457-1-AP
MRPL37 (mL37) Merck HPA025826
HSP60 Enzo Lifesciences AB1-SPA-807-E
RPUSD4 Sigma HPA039689
ERAL1 Proteintech 11478-1-AP

Secondary Antibodies
Species Manufacturer Catalog No.
Mouse Cytiva NA9310
Rabbit Cytiva NA9340

Validation

Primary Antibodies
Catalog No. Validation
10984-1-AP : Tested Reactivity: human, mouse, rat. Cited Reactivity: human, mouse.
HPA030594 : Sigma Enhanced Validation method: Independent Antibody Verification – Demonstrating antibody specificity through the use of multiple antibodies against target in IHC or ICC.
17006-1-AP : Tested Reactivity: human, mouse, rat. Cited Reactivity: human, mouse.
15682-1-AP : Tested Reactivity: human, mouse, rat. Cited Reactivity: human, mouse.
HPA050081 : Sigma Enhanced Validation method: Independent Antibody Verification – Demonstrating antibody specificity through the use of multiple antibodies against target in IHC or ICC.
16457-1-AP : Tested Reactivity: human, mouse, rat. Cited Reactivity: human, mouse.
HPA025826 : Sigma Enhanced Validation method: Independent Antibody Verification – Demonstrating antibody specificity through the use of multiple antibodies against target in IHC or ICC. Genetic Strategies – Demonstrating antibody specificity through knockout/knockdown methods. Orthogonal Validation Using RNA-seq - Demonstrating antibody specificity through an antibody-dependent method correlated with an antibody-independent method (RNA-seq).
AB1-SPA-807-E : Species Reactivity: Bovine, C. elegans, Chicken, Dog, Ehrlichia sennetsu, Guinea pig, Hamster, Human, Monkey, Mouse, Plant, Porcine, Rabbit, Rat.
HPA039689 : Sigma Enhanced Validation method: Recombinant expression.
11478-1-AP : Tested Reactivity: human. Cited Reactivity: human, mouse.

Secondary antibodies
Catalog No. Validation
NA9310, NA9340 : The antibodies are prepared by hyper-immunizing an animal with purified immunoglobulin fractions from normal human serum to produce high affinity antibodies. The pooled antiserum is used to produce an immunoglobulin preparation that is then affinity adsorbed to remove cross-reacting antibodies with the immunoglobulins of other species. These activities are thoroughly depleted to ensure species-specificity.

Eukaryotic cell lines

Policy information about cell lines and Sex and Gender in Research

Cell line source(s)

Mouse embryonic cell lines were obtained from embryos at an embryonic stage prior to physiologically observable sex differences.

Authentication

Cell lines were isolated in-house and did not require authentication.

Mycoplasma contamination

All cell lines were regularly tested for mycoplasma (approximately at 6 month intervals).

Commonly misidentified lines
(See ICLAC register)

N/A

Animals and other research organisms

Policy information about studies involving animals; ARRIVE guidelines recommended for reporting animal research, and Sex and Gender in Research

Laboratory animals	All mice were generated from a C57BL/6N strain.
Wild animals	N/A
Reporting on sex	All mouse experiements utilised tissues from both sexes.
Field-collected samples	N/A
Ethics oversight	Animal studies were approved by the local animal welfare ethics commimee (Stockholm ethical commimee) and performed in compliance with the national and European law.

Note that full information on the approval of the study protocol must also be provided in the manuscript.

Plants

Seed stocks	N/A
Novel plant genotypes	N/A
Authentication	N/A
